# Relationship of Urinary Phthalate Metabolites with Cardiometabolic Risk Factors and Oxidative Stress Markers in Children and Adolescents

**DOI:** 10.1155/2021/5514073

**Published:** 2021-04-27

**Authors:** Majid Hashemi, Mohammad Mehdi Amin, Afsane Chavoshani, Nasim Rafiei, Karim Ebrahimpour, Roya Kelishadi

**Affiliations:** ^1^Department of Environmental Health Engineering, School of Health, Isfahan University of Medical Sciences, Isfahan, Iran; ^2^Environment Research Center, Research Institute for Primordial Prevention of Non-Communicable Disease, Isfahan University of Medical Sciences, Isfahan, Iran; ^3^Environmental Health Engineering, School of Public Health, Kerman University of Medical Sciences, Kerman, Iran; ^4^Environmental Health Engineering Research Center, Kerman University of Medical Sciences, Kerman, Iran; ^5^Child Growth and Development Research Center, Research Institute for Primordial Prevention of Non-Communicable Disease, Isfahan University of Medical Sciences, Isfahan, Iran

## Abstract

**Introduction:**

Studies have proved that exposure of adults to phthalates might be related to cardiometabolic risk factors and changes in markers of oxidative stress. Such studies conducted on school-age children and adolescents are limited and fail to assess the simultaneous effect of phthalates on these risk factors and oxidative stress markers. Therefore, it was attempted to identify the relationship of urinary phthalate metabolites with cardiometabolic risk factors and oxidative stress markers in children and adolescents*. Methods*. In this cross-sectional study, 108 children and adolescents, living in Isfahan industrial city of Iran, were examined. Urine samples taken from the participants were analyzed for mono-butyl phthalate (MBP), mono-benzyl phthalate (MBzP), mono-(2-ethylhexyl) phthalate (MEHP), mono-(2-ethyl-5-hydroxyhexyl) phthalate (MEHHP), mono-(2-ethyl-5-exohexyl) phthalate (MEOHP), and mono-methyl phthalate (MMP).

**Results:**

Results showed that, among phthalate metabolites, MBP had the highest concentration, followed by MBzP, MEOHP, MEHHP, MEHP, and MMP. Concentrations of these metabolites had a significant relationship with some of the cardiometabolic risk factors including systolic blood pressure (SBP), fasting blood sugar (FBS), and triglycerides (TG) (*p* < 0.05). Furthermore, the crude and adjusted linear regression models indicated the significant association of phthalate metabolites with superoxide dismutase (SOD), malondialdehyde (MDA), and homeostasis model assessment of insulin resistance (HOMA-IR) (*p* < 0.05).

**Conclusion:**

Although urinary phthalate concentrations could not exactly reflect the long-term exposure level in the studied age groups, the consumption of phthalate-free products during childhood and adolescent development shall be assumed helpful in maintaining a healthy lifestyle. To confirm these findings and develop effective intervention strategies, it would be necessary to perform longitudinal studies on diverse population.

## 1. Introduction

Changes in diet and lifestyle have increased the incidence and prevalence of the health disorders associated with emerging micropollutants [1]. Phthalic acid esters (phthalates), as priority pollutants and endocrine disruptive compounds (EDCs), are the synthetic chemicals widely used as plasticizers in children's toys, hygiene and cosmetics products, medical equipment, food packaging and processing, building materials, and floorings [2–5]. Due to the widespread use, phthalates can increase the chance of infertility, primary ovarian insufficiency, and other abnormalities of the female reproductive system [2, 6–9]. They can also lead to disorders of hormonal signaling pathways of the thyroid, immune, and metabolic systems [10–12]. The US Environmental Protection Agency (EPA) classified di-(2-ethylhexyl) phthalate (DEHP) and benzylbutyl phthalate (BzBP) as potential human carcinogens, respectively [10]. Exposure to phthalates may occur via oral ingestion, inspiration, and dermal contact [13–16]. Based on molecular weight, phthalates may be classified into two groups including low-molecular-weight phthalates (with carbon chains of four or less) and high-molecular-weight phthalates (with carbon chains more than four). The major sources of human exposure to low and high molecular weight phthalates are personal care products and diet, respectively [10]. In a human body, phthalates are quickly converted into their monoesters excreted in the urine either freely or conjugated as glucuronides. Therefore, human exposure to phthalates is measured by urinary phthalate metabolites [17–19]. Exposure to phthalates can influence the cardiometabolic risk factors (obesity, hypertension, blood sugar level, high triglyceride levels, and low- and high-density lipoprotein levels) [20]. Moreover, a direct relationship has been observed between phthalate exposure and increase of oxidative stress markers [21]. Many studies have provided strong evidence on the relationship between phthalate exposure and higher risk of insulin resistance syndrome [21–27]. The relationship between phthalate concentration and insulin resistance syndrome can be evaluated through homeostasis model assessment of insulin resistance (HOMA-IR), which is known as an alternative useful technique [21, 28–30]. Higher HOMA-IR concentrations have been observed among the elderly participants exposed to DEHP metabolites [31]. Oxidative stress, known as an imbalance between the amount of free oxidative radicals and antioxidants, can adversely alter lipid peroxidation, protein oxidation, and DNA oxidation [21, 32]. Moreover, inappropriate production of other oxidative stress markers, such as malondialdehyde (MDA) and superoxide dismutase (SOD), can occur under exposure to DEHP concentration [33]. Previous studies showed a positive correlation between phthalates exposure and MDA concentration [34–36]. Both DEHP and monobutyl phthalate (MnBP) exposures were involved in increase of oxidative stress markers and fatty acid oxidation [37].

Since cardiometabolic and oxidative stress markers, such as SOD, MDA, and HOMA-IR, are related to phthalates exposure, it is assumed that children and adolescents are more susceptible to phthalates exposure. To the best of the author's knowledge, no study yet has been conducted on the simultaneous effect of phthalates on cardiometabolic and oxidative stress markers. Therefore, the purpose of this study is to identify the relationships of phthalates with cardiometabolic risk factors and oxidative stress markers among children and adolescents.

## 2. Materials and Methods

A cross-sectional study was conducted among 108 children and adolescents aged 6–18 years living in Isfahan industrial city, Iran. It was approved by the Research and Ethics Committee of Isfahan University of Medical Sciences (project code: 394984). The participants gave an oral assent and their parents signed a form for informed consent. Demographic characteristics of the participants were collected using a validated questionnaire. To reduce the effect of recall bias, the questionnaires were completed jointly by parents and their children. Accordingly, physical examinations, including measurements of weight, height, waist circumference, diastolic blood pressure (DBP), and systolic blood pressure (SBP), were conducted using the standards protocols [38]. Moreover, the body mass index (BMI) was calculated as wt (kg) divided by height squared (m^2^). Other cardiometabolic risk factors, such as fasting blood sugar (FBS), triglycerides (TG), high-density lipoprotein cholesterol (HDL-C), low-density lipoprotein cholesterol (LDL-C), and total cholesterol (TC), were determined by standard kits (Pars Azmoun, Tehran) and automatic analyzers [38]. The abnormality levels of the measured clinical parameters were TC ≥ 200 mg/dL; TG ≥ 100 mg/dL for children below nine years old and TG ≥ 130 mg/dL for children aged 10–18 years; HDL-C < 40 mg/dL; LDL-C ≥ 130 mg/dL; FBS ≥ 100 mg/dL. Serum MDA and SOD were measured by Elisa Kits. Insulin resistance (IR) was calculated according to (1) [39].

(1) $$\begin{array}{c} \text{HOMA}-\text{IR}=\frac{\text{fasting glucose}\left(\text{mg}\cdot {\text{dl}}^{-1}\right)\times \text{fasting insulin}\left(\mu {\text{UmL}}^{-1}\right)}{405}. \end{array}$$

The abnormality level for HOMA-IR is > 2.5.

The standard stock solutions of phthalates (MEHP, MMP, MBzP, MBP, MEOHP, and MEHHP) were prepared by adding methanol, poured into glass bottles to avoid contamination, and stored at a temperature of less than 0°C. Concentrations of 0.001, 0.005, 0.01, 0.05, 0.1, and 0.5 *μ*g/mL of the aforementioned metabolites were used to draw the calibration curve. Urine samples were thawed at room temperature before analysis. Afterwards, 5 ml of the urine samples was poured into a glass test tube made of borosilicate, and 20 *μ*L of *β*-glucuronidase enzyme was added to digest the samples and remove the compounds from the glucuronide state. The samples were incubated in a shaker (37°C for 18 h). After removing the samples from the shaker incubator, they were diluted and their pH was adjusted to 2 by 10% sulfuric acid. Different amounts of acetonitrile (as a disperser) and chlorobenzene (as an extractant) were used to extract the phthalate metabolites by dispersive liquid-liquid microextraction (DLLME) method. After several trials and errors, the best extraction was obtained using 750 *μ*L of acetonitrile and 80 *μ*L of chlorobenzene. Chlorobenzene and acetonitrile were rapidly injected into the samples to form a cloudy solution. The samples were then placed in a centrifuge (at 6000 rpm for 5 m). The precipitate at the bottom of the tube (20 *μ*L) was extracted by a syringe and dried by nitrogen gas. After adding N-methyl-N-(trimethylsilyl) trifluoroacetamide (MSTFA) derivative to the obtained substance, it was injected into a gas chromatography/mass spectrometry (GC/MS) system [38]. A gas chromatography (GC) device (Agilent, model: 7890A) equipped with a mass selective detector (MSD) (Agilent, model: 5975C) made by Agilent technology was used to measure the amount of urinary phthalate metabolites, and ChemStation software under Windows XP was used to operate the gas chromatography/mass selective detector (GC/MSD) device. Moreover, DB5-MS column produced by Agilent company (with a length of 60 m, column diameter of 25 mm, and internal film of 25*µ*) and helium (grade 5.5) as a carrier gas with a flow rate of 1 mL/min were applied. The injection port (injector) at 270°C was used as a split with a ratio of 2 : 1 to inject the sample. The oven temperature started with 100°C (initial temperature) and remained at 100°C for 3 m. Then, it reached 300°C with a slope of 20°C/min and was kept at the same temperature for 7 m. The ion source temperature and transfer line temperature (transfer line) were set at 230°C and 290°C, respectively. Finally, the MSD was set to selected ion monitoring (SIM) mode. The ions used for SIM, retention time, and time window for the studied metabolites are presented in Table 1.

### 2.1. Method Validation

Recovery at three concentrations of low, medium, and high was evaluated using (2). Limit of detection (LOD) and limit of quantitation (LOQ) for each phthalate monoester were calculated using 3*S*_0_ and 10*S*_0_, respectively. *S*_0_ is the value of the standard deviation obtained through blank analysis (in this study: 15 blank analyses). In this study, all values below the LOD were replaced by one-half of the LOD [38].

(2) $$\begin{array}{c} R=\frac{C}{C_{\text{ref}}}*100, \end{array}$$

where *R* is recovery; *C* is measurement concentration; *C*_ref_ is reference concentration.

Relative standard deviation (RSD) is a standard deviation of a set of data divided by the mean of the data and can be calculated by

(3) $$\begin{array}{c} \text{RSD }\%=\frac{S}{X_{\text{ave}}}*100, \end{array}$$

where *S* is standard deviation and *X*_ave_ is the mean.

### 2.2. Statistical Analysis

Statistical analyses were performed using SPSS software (version 25.0, IBM, USA) and Graph-Pad Prism® 8.0 software (USA). An independent sample *t*-test was used to compare the means of cardiometabolic risk factors (age, obesity, waist, SBP, DBP, FBS, LDL-C, HDL-C, TC, and TG) and oxidative stress markers (SOD, MDA, and HOMA-IR). Linear regression was also used to estimate the relationships of phthalate concentrations with cardiometabolic risk factors, three tertiles of oxidative stress markers, and exposure sources. In the adjusted model, the relationships between the phthalate metabolites and oxidative stress markers were adjusted based on gender, age, BMI, SBP, DBP, FBS, TC, and TG variables. However, due to the high correlation of these variables with other variables and probability of multicollinearity error occurrence, waist, HDL-C, and LDL-C were excluded from the analysis. It should be noted that *p*-values < 0.05 were considered as statistically significant.

## 3. Results

### 3.1. Validation of Results

Validation of results indicated the high accuracy of the method (Table 2). Recovery was evaluated at three concentrations: low, medium, and high. According to Table 2, recoveries of MEHP, MEOHP, MEHHP, MMP, MBP, and MBzP metabolites in three different concentrations were 55–68, 90–96, 69–109, 86–77, 93–83, and 102–93, respectively. The LOD and LOQ calculated for each monoester are presented in Table 2 [40].

### 3.2. Characteristics of the Studied Population

Among the participants' demographic characteristics, including age group, parents' education level and employment status, physical activity, shower times per week, toy materials preference (plastic or metals), cosmetic consumption, plastic packaging use, and bottled beverage use, only mothers' employment status (*p* = 0.034) and toy materials preference (*p* = 0.008) had significant differences among children and adolescents (Table 3).

### 3.3. Clinical and Physical Examinations

Table 4 shows the participants' clinical and physical examination results based on mean and standard deviation (SD). Overall, the participants, with a mean (SD) age of 11.63 (2.31) years, were composed of 57 girls (57.9%) and 51 boys (42.1%). According to BMI, 49 (54%), 40 (37%), and 19 (17.6%) of the participants were considered normal, overweight, and obese, respectively. The participants' mean (SD) waist circumference was 85.57 (12.07) cm, with the boys and the girls having the mean (SD) waist circumferences of 89.29 (10.64) cm and 82.24 (12.38) cm, respectively. SBP and DBP for the whole participants were 11.28 (1.28) and 6.85 (0.69) mmHg, respectively. As shown in Table 4, the means (SD) of TC, HDL-C, LDL-C, and TG in the participants were 171.33 (33.08), 48.38 (12), 102.85 (25.76), and 108.63 (60.34) mg/dL, respectively. Among the participants, 17.6% had abnormal TC, 64.8% had abnormal triglycerides, 20.37% had abnormal HDL-C, 13.88% had abnormal LDL-C, 89% had normal FBS, and 11% had impaired fasting glucose (IFG). Moreover, the means (SD) of SOD, MDA, and HOMA-IR in the participants were 152 (69.96), 21.95 (9.23), and 2.66 (1.86), respectively, and 37% of the children and adolescents had abnormal HOMA-IR. The mean (SD) concentrations of MMP, MBP, MEHP, MBzP, MEOHP, and MEHHP were 63.09 (25.96), 260.46 (158.26), 133.09 (133.46), 249.51 (186.88), 249.16 (142.47), and 210.37 (138.80) *μ*g/L, respectively. Among the metabolites of phthalates, MBP had the highest concentration, followed by MBzP, MEOHP, MEHHP, MEHP, and MMP.

### 3.4. Phthalates and Cardiometabolic Risk Factors

**Figure 1:**
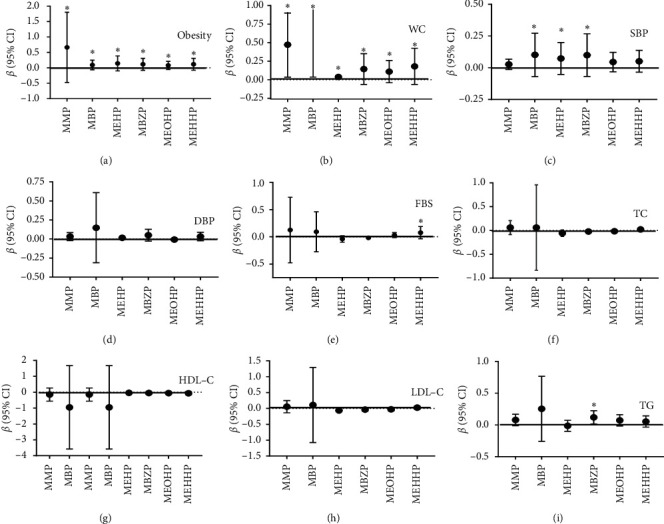
The association between urinary phthalates and cardiometabolic risk factors ((a) obesity status, (b) WC, (c) SBP, (d) DBP, (e) FBS, (f) TC, (g) HDL-C, (h) LDL-C, (i) TG) in children and adolescents. ^*∗*^*p* < 0.05.

Figure 1 shows the relationships between each phthalate metabolite and cardiometabolic risk factors. Statistically, the significant relationships were observed among the phthalate metabolites, obesity status, and waist circumference (*p* < 0.05) (Figures 1(a) and 1(b)). A positive relationship was observed between SBP and MBP [*β*: 0.299, CI: (0.001, 0.004), *p* = 0.002], SBP and MEHP (*β* = 0.218, CI: (0.001, 0.001), *p* = 0.023] and SBP and MB_Z_P [*β*: 0.293, CI: (0.001, 0.003), *p* = 0.002]. Likewise, there was a significant relationship between FBS and MEHHP [*β*: 0.205, CI: (0.001, 0.023), *p* = 0.034] (Figure 1(c)). However, an insignificant relationship was found among the phthalate metabolites, DBP, TC, HDL-C, and LDL-C (*p* > 0.05) (Figures 1(d)–1(h)). Serum TG was related to the MB_Z_P concentration [*β*: 0.217, CI: (0.009, 0.131), *p* = 0.024] (Figure 1(i)).

### 3.5. Phthalate Metabolites and Oxidative Stress Markers (SOD, MDA, and HOMA-IR)

The relationships between the phthalate metabolites and tertiles of oxidative stress markers (SOD, MDA, and HOMA-IR) have been presented in Table 5. In the middle tertile, the crude and adjusted models showed that one-unit increase in MEOHP concentration led to 0.03 decrease (*p* = 0.040) and 0.044 decrease (*p* = 0.015) in SOD concentration, respectively. In the highest tertile, in the adjusted model, one-unit increase in MEOHP concentration led to 0.282 decrease in SOD concentration. Moreover, the 2^nd^ and the 3^rd^ tertiles of the crude and adjusted models revealed that all the phthalate metabolites, except MEHP, had a negative relationship with MDA (*p* < 0.05). In the crude model, one-unit increase in MMP, MBP, MBzP, MEOHP, and MEHHP led to 0.006–0.029 decrease in MDA concentration (*p* < 0.05). However, this decrease was 0.005–0.028 in the adjusted model (*p* < 0.05). In an analysis stratified by HOMA-IR in the highest tertile, MMP in the crude model (*p* = 0.049) and MBP in the adjusted model (*p* = 0.046) had negative relationships with HOMA-IR.

### 3.6. Phthalate Concentrations and Health Behaviors

Table 6 indicates that MEOHP has inverse (*β* = -63.44, *p* = 0.031) and positive (*β* = 79.15, *p* = 0.01) relationships with moderate physical activity and shower times per week, respectively.

## 4. Discussion

The evaluation of the demographic variables revealed that mothers' employment status and toy materials preference had significant differences among boys and girls (*p* < 0.05). Demographic variables are the most important predictors for health and illness of girls and boys in childhood and adolescence [41]. For instance, age and gender are important variables for exposure to the endocrine-disrupting chemicals (EDCs) in the environment. Buser et al. reported a significant positive relationship between high molecular weight (HMW) phthalates and male gender. Their results showed an increased odd of being obese among the adults exposed to HMW phthalate metabolites [42]. Won et al. observed that age and gender had significant relationships with urinary phthalate concentrations. They found that 6–11-year-old children were more susceptible to phthalate concentrations, which might lead to neurobehavioral development [41]. In the current study, we found no relationship among concentrations of phthalate metabolites, age, and gender. This could be due to the younger age of the participants we studied. The simple linear regression analyses showed that MMP, MBP, MEHP, MB_Z_P, MEOHP, and MEHHP metabolites had significant positive relationships with measures of generalized and abdominal obesity. In fact, adipose tissue or body fat acts as an endocrine organ and can be the prime target for endocrine disruptions. Under exposure to EDCs, such as phthalates, this organ can improperly secrete numerous chemical signals or might have an inappropriate response to them [43].

Our findings were in agreement with the results obtained by Wang et al., who reported a positive relationship among urinary phthalate concentrations, waist circumference, and BMI in the Chinese schoolchildren. This relationship remained significant after making adjustments for age and gender [44]. Likewise, Teitelbaum et al. identified a positive relationship between urinary monoethyl phthalate (MEP) concentration and BMI among overweight children [45]. In contrast, Boas et al. observed that some phthalate metabolites, such as MEP, MEOHP, and mono-(2-ethyl-5-carboxypentyl) phthalate (MECPP), had an inverse correlation with BMI in the Danish children aged four to nine years [46]. In an experimental study, Zhou et al. reported that, compared with control, weight gain in rats was significantly increased after exposure to DEHP [47].

The present study indicated the relationships of all phthalates with obesity. It also showed that MBP and MBzP metabolites were associated with SBP but not DBP. Moreover, MB_Z_P and MEHHP were associated with elevated TG and FBS, respectively. Baralic et al. demonstrated that a mixture of low doses of DEHP, DBP, and BPA could induce significant changes in weight gain, appetite rate, lipid profile, glucose level, and hormonal concentrations in various glands [48]. Blood pressure (BP) has been extensively studied for phthalate implications [49]. For example, it has been found that exposures to DEHP and MEP metabolites were associated with lower SBP (but not DBP) in the girls aged four to seven years [50]. Su et al. showed that BMI, DBP, BP, diabetes mellitus, FBS, and TG were associated with increased levels of MEHP among young people, which is in agreement with our findings [51]. Another study demonstrated that phthalate compounds had positive relationships with BP, but not TG or HDL, in children and adolescents [52]. According to Poursafa et al., obesity, elevated BP, hyperglycemia, and dyslipidemia are among the leading risk factors for noncommunicable diseases (NCDs), whose origins can be traced back in childhood [53].

We found that, among phthalates, MEOHP had a negative relationship with SOD levels (*p* < 0.05), and MMP and MBP metabolites had negative relationships with HOMA-IR of children and adolescents (*p* < 0.05). Previous studies revealed that, after exposure to phthalate metabolites, MDA content was increased in the infants and children undergoing cyclic parenteral nutrition [34]. However, in the current study, the relationships between MDA and all phthalates, except MEHP, were negatively significant. This might be attributed to two factors: (1) high antioxidants capacity of adolescents and children due to probable use of dietary supplements, such as alpha-lipoic acid (ALA), for weight loss or other treatment purposes, and (2) stimulation of body protective defense responses to the oxidative stress neutralization under exposure to phthalate compounds. It has been already proved that ALA administration to the phthalate-treated mice could significantly reduce MDA concentrations compared with phthalate group [54]. Yang et al. also found that 6-gingerol, as an antioxidant, could prevent the oxidative stress induced by MEHP [55]. We found that physical activity and shower times per week had a significant relationship with urinary concentration of MEOHP metabolite. Since phthalates are used in the personal care products, shower times per week were considered as a source of exposure to phthalates among children and adolescents. Moreover, systemic absorption of VOCs was partially increased in young children during the physical activities (as a source of exposure). Respiratory rates and cardiac outputs observed in infants and young children were relatively high and favored the uptake of inhaled VOCs [56].

The main limitation of the current study stems from the cross-sectional nature of the data, which impeded the study of the cause-effects of variables. Moreover, we failed to examine the pubertal status of the participants, which might affect the variables studied. However, novelty in choosing the pediatric age group, determining various metabolites of phthalates, and considering the simultaneous effect of phthalates on cardiometabolic risk factors and oxidative stress are the main strengths of this study.

## 5. Conclusion

The results indicated the relationships of all the studied phthalate metabolites with generalized and abdominal obesity. They asserted the necessity of considering the exposure to phthalates as an emerging risk factor for obesity among children and adolescents. We also detected a significant relationship between SBP and MBP, MEHP and MBzP metabolites; FBS and MEHHP metabolite; TG and MBzP metabolite. Moreover, most of the phthalates had a significant relationship with SOD, MDA, and HOMA-IR. Due to the hydrophobic feature of phthalate and wide range of ages in children and adolescents, urinary phthalate concentrations could not exactly reflect the long-term exposure level and severity of cardiometabolic diseases. However, still it seemed more cautious to consume phthalate-free products during childhood and adolescent development in order to maintain a healthy lifestyle. However, longitudinal studies on diverse population seem necessary to confirm these findings and develop the effective interventions strategies.

## Figures and Tables

**Table 1:** Chemical structure, retention time, selected ions, and time window for the analyzed analytes (MEHP, MMP, MBzP, MBP, MEOHP, and MEHHP).

Metabolites	The chemical structure	Time separation (minutes)	Selected ions	Time window
MEHP	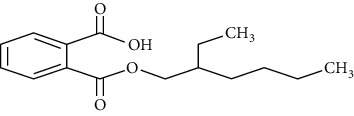	14.7	149, 221, 239	14–15.1
MEOHP	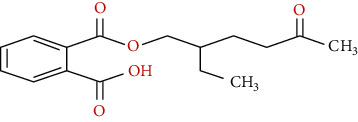	15.8	108, 127, 149, 221, 239	15.6–15.95
MEHHP	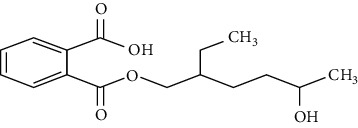	16.05	117, 147, 221, 265, 295	15.95–20
MMP	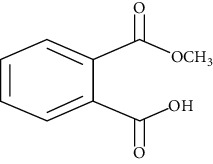	11.8	89, 237, 163	11.3–12
MBP	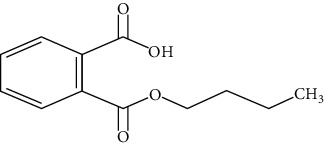	13.2	223, 149, 163	12–13.8
MBzP	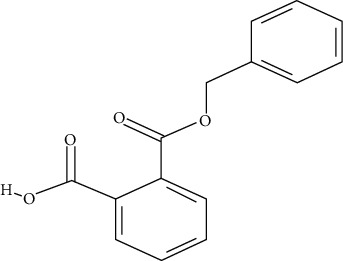	15.4	91, 179, 222	14.9–15.6

**Table 2:** Validation results of the method to identify the studied metabolites.

Metabolites	LOD	LOQ	Recovery			RSD (%)	*R* ^2^
			Low concentration (1 *µ*/L)	Medium concentration (10 *µ*/L)	High concentration (100 *µ*/L)		
MEHP	0.024	0.05	90	55	69	13.2	0.997
MEOHP	0.23	0.48	80	68	96	8.5	0.997
MEHHP	0.088	0.18	69	109	105	6.2	0.995
MMP	0.03	0.1	77	79	86	5.6	0.993
MBP	0.36	1.2	83	89	93	7	0.997
MBzP	0.54	1.8	94	93	102	6.9	0.991

**Table 3:** Participant's demographic and behavioral characteristics.

	Girl	Boy	Total	*p*-value
	*n* (%)	*n* (%)	*n* (%)	
*Age groups*				
6–11 years	28 (49.1)	17 (33.3)	45 (41.66)	0.098
12–18 years	29 (50.9)	34 (66.7)	63 (58.34)	
*Fathers' education level*				
Illiterate	4 (7)	2 (3.9)	6 (5.55)	0.338
No college education	44 (77.2)	38 (74.5)	82 (75.9)	
College education	9 (15.8)	11 (21.6)	20 (18.55)	
*Mothers' education level*				
Illiterate	1 (1.8)	1 (2)	2 (1.85)	0.249
No college education	46 (80.7)	38 (70.6)	84 (77.77)	
College education	10 (17.5)	14 (27.5)	24 (20.38)	
*Fathers' job*				
Unemployed	10 (17.5)	6 (11.8)	16 (14.81)	0.585
Employed	10 (17.5)	10 (19.6)	20 (18.51)	
Self-employment	37 (64.9)	35 (68.6)	72 (66.68)	
*Mothers' job*				
Unemployed	46 (80.7)	48 (94.1)	94 (87)	0.034
Employed	7 (12.3)	3 (5.9)	10 (9.25)	
Self-employment	4 (7)	—	4 (3.75)	
*Physical activity*				
Low	29 (50.9)	23 (45.1)	52 (48.14)	0.638
Moderate	19 (33.3)	20 (39.2)	39 (36.11)	
High	9 (15.8)	8 (15.7)	17 (15.75)	
*Shower times per week*				
2 times	35 (61.4)	25 (49)	60 (55.5)	0.306
3 times	12 (21.1)	17 (33.3)	29 (26.85)	
>4 times	10 (17.5)	9 (17.6)	19 (18)	
*Toy materials*				
Plastic	51 (89.5)	35 (68.6)	86 (79.62)	0.008
Metallic	6 (10.5)	16 (31.4)	22 (20.38)	
*Cosmetic consumption*				
Yes	51 (89.5)	46 (90.2)	97 (89.81))	0.902
No	6 (10.5)	5 (9.8)	11 (10.9)	
*Plastic packing use*				
Yes	36 (63.2)	28 (54.9)	64 (59.25)	0.386
No	21 (36.8)	23 (45.1)	44 (40.75)	
*Bottled beverage use*				
Low	45 (80.7)	38 (74.5)	83 (76.85)	0.415
Moderate	11 (19.3)	12 (23.5)	23 (21.29)	
High	0	1 (20)	1 (1.86)	

**Table 4:** Clinical and physical examinations result of the participants.

		All subjects (*n* = 108)	Girls (*n* = 57)	Boys (*n* = 51)
BMI; *n* (%)	Normal	49 (45.4)	32 (56.1)	17 (33.3)
	Overweight	40 (37)	18 (31.6)	22 (43.1)
	Obese	19 (17.6)	7 (12.3)	12 (23.5)
Age; mean (SD)		11.63 (2.31)	11.39 (2.45)	11.91 (2.13)
Waist; mean (SD)		85.57 (12.07)	82.24 (12.38)	89.29 (10.64)
SBP; mean (SD)		11.28 (1.28)	10.94 (1.27)	11.65 (1.20)
DBP; mean (SD)		6.85 (0.69)	6.82 (0.71)	6.88 (0.68)
FBS; mean (SD)		89.78 (8.28)	88.33 (8.84)	91.39 (7.34)
TC; mean (SD)		171.33 (33.08)	164.95 (29.78)	178.47 (35.35)
HDL-C; mean (SD)		48.38 (12)	46.85 (10.11)	50.09 (13.71)
LDL-C; mean (SD)		102.85 (25.76)	98.24 (23.87)	108 (27.02)
TG; mean (SD)		108.63 (60.34)	109 (56.37)	108.22 (65.07)
SOD; mean (SD)		152 (69.96)	150.88 (63)	153.5 (76.84)
MDA; mean (SD)		21.95 (9.23)	22.22 (9.3)	21.67 (9.27)
HOMA-IR, mean (SD)		2.66 (1.86)	2.68 (1.93)	2.22 (1.77)
MMP; mean (SD)		63.09 (25.96)	63.10 (24.86)	63.07 (27.38)
MBP; mean (SD)		260.46 (158.26)	252.40 (166.3)	269.47 (149.89)
MEHP; mean (SD)		133.09 (133.46)	120.04 (120.71)	147.67 (146.25)
MBzP; mean (SD)		249.51 (186.88)	225.68 (171.15)	276.15 (201.39)
MEOHP; mean (SD)		249.16 (142.74)	228.33 (137.76)	272.44 (145.95)
MEHHP; mean (SD)		210.37 (138.80)	193.48 (146.54)	229.25 (128.39)

**Table 5:** The association between exposure to urinary phthalates and tertiles of oxidative stress markers in children and adolescent.

	Tertile 1				Tertile 2				Tertile 3			
	Crude		Adjusted		Crude		Adjusted		Crude		Adjusted	
	*β* (S.E)	*p*	*β* (S.E)	*p*	*β* (S.E)	*p*	*β* (S.E)	*p*	*β* (S.E)	*p*	*β* (S.E)	*p*
**SOD**	**<116**				**116–162**				**>162**			
MMP	−0.060 (0.075)	0.428	−0.017 (0.075)	0.825	−0.098 (0.097)	0.311	−0.047 (0.125)	0.712	0.307 (0.505)	0.544	0.463 (0.638)	0.469
MBP	0.004 (0.011)	0.717	0.018 (0.014)	0.189	0.003 (0.015)	0.828	0.009 (0.022)	0.691	0.037 (0.089)	0.677	−0.016 (0.121)	0.897
MEHP	−0.008 (0.019)	0.699	−0.023 (0.023)	0.333	−0.010 (0.018)	0.592	−0.037 (0.026)	0.156	0.051 (0.090)	0.576	−0.001 (0.116)	0.993
MBZP	0.002 (0.012)	0.894	0.019 (0.015)	0.216	0.006 (0.013)	0.633	0.002 (0.017)	0.905	0.030 (0.074)	0.690	−0.027 (0.101)	0.790
MEOHP	−0.003 (0.014)	0.820	0.001 (0.014)	0.946	**−0.030 (0.014)**	**0.040**	**−0.044 (0.015)**	**0.004**	−0.167 (0.100)	0.097	**−0.282 (0.095)**	**0.003**
MEHHP	−0.011 (0.014)	0.445	−0.014 (0.015)	0.355	−0.011 (0.016)	0.500	−0.007 (0.020)	0.744	−0.056 (0.118)	0.636	−0.303 (0.157)	0.055
**MDA**	**<16**				**16–24**				**>24**			
MMP	−0.006 (0.012)	0.640	0.002 (0.012)	0.867	**−0.029 (0.014)**	**0.043**	**−0.028 (0.014)**	**0.042**	0.133 (0.068)	0.053	0.033 (0.090)	0.716
MBP	−0.002 (0.002)	0.313	−0.001 (0.002)	0.534	**−0.006 (0.003)**	**0.015**	**−0.008 (0.003)**	**0.003**	0.001 (0.012)	0.968	−0.020 (0.010)	0.061
MEHP	−0.002 (0.003)	0.594	−0.004 (0.005)	0.452	−0.002 (0.003)	0.421	−0.002 (0.003)	0.507	0.023 (0.012)	0.057	−0.003 (0.012)	0.798
MBZP	−0.001 (0.002)	0.465	−0.003 (0.002)	0.151	−0.004 (0.002)	0.135	**−0.007 (0.003)**	**0.030**	**−0.019 (0.009)**	**0.036**	**−0.025 (0.010)**	**0.017**
MEOHP	−0.001 (0.002)	0.667	−0.001 (0.002)	0.585	−0.004 (0.002)	0.124	**−0.005 (0.003)**	**0.044**	−0.008 (0.013)	0.556	**−0.031 (0.010)**	**0.003**
MEHHP	0.001 (0.002)	0.960	0.001 (0.002)	0.627	**−0.006 (0.002)**	**0.023**	**−0.013 (0.003)**	**<0.001**	0.005 (0.015)	0.713	−0.027 (0.013)	0.053
**HOMA-IR**	**<1.55**				**1.55–2.66**				**>2.66**			
MMP	−0.001 (0.003)	0.636	0.002 (0.003)	0.385	0.001 (0.002)	0.792	0.004 (0.002)	0.060	**−0.021 (0.010)**	**0.049**	−0.021 (0.012)	0.085
MBP	<0.001 (0.001)	0.327	<0.001 (0.001)	0.163	<0.001 (0.001)	0.940	<0.001 (0.001)	0.446	−0.002 (0.002)	0.241	**−0.005 (0.002)**	**0.046**
MEHP	<0.001 (0.001)	0.435	<0.001 (0.001)	0.749	<0.001 (0.001)	0.860	<0.001 (0.001)	0.764	−0.001 (0.002)	0.606	−0.002 (0.003)	0.521
MBZP	<0.001 (0.001)	0.208	<0.001 (0.001)	0.381	<0.001 (0.001)	0.331	<0.001 (0.001)	0.119	<0.001 (0.001)	0.803	−0.001 (0.002)	0.682
MEOHP	−0.001 (0.001)	0.055	<0.001 (0.001)	0.812	<0.001 (0.001)	0.725	<0.001 (0.001)	0.427	−0.003 (0.002)	0.241	−0.004 (0.002)	0.077
MEHHP	<0.001 (0.001)	0.733	<0.001 (0.001)	0.892	<0.001 (0.001)	0.323	<0.001 (0.001)	0.870	−0.001 (0.002)	0.700	−0.001 (0.003)	0.795

The linear regression results were adjusted by gender, age, BMI, SBP, DBP, FBS, TC, and TG variables.

**Table 6:** Association between phthalate concentrations with health behaviors based on multiple linear regression.

Health behaviors	MMP		MBP		MEHP		MBZP		MEOHP		MEHHP	
	*β* (S.E)	*p*	B (S.E)	*p*	*β* (S.E)	*p*	*β* (S.E)	*p*	*β* (S.E)	*p*	*β* (S.E)	*p*
*Physical activity*												
Low (*n* = 52)	Ref.											
Moderate (*n* = 39)	−6.291 (5.422)	0.246	−43.93 (33.09)	0.184	−27.25 (27.91)	0.329	−27.65 (39.25)	0.481	**−63.44 (29.46)**	**0.031**	−22.82 (29.10)	0.433
High (*n* = 17)	2.924 (7.151)	0.683	−12.66 (43.64)	0.772	−42.87 (36.81)	0.244	15.20 (51.77)	0.769	−34.69 (38.86)	0.372	−37.00 (38.38)	0.335
*Shower times per week*												
2 times (*n* = 60)	Ref.											
3 times (*n* = 29)	8.378 (5.779)	0.147	10.94 (35.60)	0.759	22.44 (29.90)	0.453	12.42 (41.98)	0.767	**79.15 (30.75)**	**0.010**	36.79 (30.98)	0.235
>4 times (19)	6.268 (6.727)	0.351	−2.401 (41.44)	0.954	−15.38 (34.80)	0.659	−24.12 (48.86)	0.622	−36.14 (35.80)	0.313	−10.39 (36.06)	0.773
*Toy materials*												
Plastic (*n* = 86)	Ref.											
Metallic (*n* = 22)	7.304 (6.133)	0.234	55.31 (37.25)	0.138	−0.241 (31.73)	0.994	39.28 (44.28)	0.375	70.43 (33.26)	0.054	50.92 (32.64)	0.119
*Cosmetic consumption*												
Yes (*n* = 97)	Ref.											
No (*n* = 11)	−3.173 (8.214)	0.699	−22.71 (50.06)	0.650	−46.47 (42.02)	0.269	−63.98 (58.85)	0.277	−3.079 (45.20)	0.946	31.71 (43.84)	0.469
*Special drug*												
Yes (*n* = 19)	Ref.											
No (*n* = 89)	2.149 (6.526)	0.742	27.24 (39.72)	0.493	−21.51 (33.50)	0.521	37.23 (46.87)	0.427	10.59 (35.89)	0.768	−4.965 (34.91)	0.887
*Plastic packing use*												
Yes (*n* = 64)	Ref.											
No (*n* = 44)	−2.396 (5.054)	0.635	6.996 (30.84)	0.821	0.756 (26.01)	0.977	12.24 (36.40)	0.737	−17.61 (27.77)	0.526	40.21 (26.77)	0.133
*Bottled beverage use*												
Low (*n* = 84)	Ref.											
Moderate (*n* = 23)	8.722 (5.971)	0.144	6.663 (37.05)	0.858	13.68 (31.23)	0.661	27.55 (43.44)	0.526	6.500 (33.32)	0.845	37.31 (32.23)	0.247
High (*n* = 1)	**—**	**—**	**—**	**—**	**—**	**—**	**—**	**—**	**—**	**—**	**—**	**—**

## Data Availability

The data used in this paper are available at https://pubmed.ncbi.nlm.nih.gov/30092535/ (Phthalates Implications in the Cardiovascular System).
